# Sparse Robot Swarms: Moving Swarms to Real-World Applications

**DOI:** 10.3389/frobt.2020.00083

**Published:** 2020-07-02

**Authors:** Danesh Tarapore, Roderich Groß, Klaus-Peter Zauner

**Affiliations:** ^1^School of Electronics and Computer Science, University of Southampton, Southampton, United Kingdom; ^2^Department of Automatic Control and Systems Engineering, The University of Sheffield, Sheffield, United Kingdom

**Keywords:** swarm robotics, multirobot systems, field robotics, forest robots, sparse coupling, communication networks, information propagation, long-range radio

## Abstract

Robot swarms are groups of robots that each act autonomously based on only local perception and coordination with neighboring robots. While current swarm implementations can be large in size (e.g., 1,000 robots), they are typically constrained to working in highly controlled indoor environments. Moreover, a common property of swarms is the underlying assumption that the robots act in close proximity of each other (e.g., 10 body lengths apart), and typically employ uninterrupted, situated, close-range communication for coordination. Many real world applications, including environmental monitoring and precision agriculture, however, require scalable groups of robots to act jointly over large distances (e.g., 1,000 body lengths), rendering the use of *dense* swarms impractical. Using a dense swarm for such applications would be invasive to the environment and unrealistic in terms of mission deployment, maintenance and post-mission recovery. To address this problem, we propose the *sparse* swarm concept, and illustrate its use in the context of four application scenarios. For one scenario, which requires a group of rovers to traverse, and monitor, a forest environment, we identify the challenges involved at all levels in developing a sparse swarm—from the hardware platform to communication-constrained coordination algorithms—and discuss potential solutions. We outline open questions of theoretical and practical nature, which we hope will bring the concept of sparse swarms to fruition.

## 1. Introduction

Swarm robotics takes inspiration from observed behaviors of collective systems in nature (Camazine et al., 2003) to develop large-scale teams of robots with limited individual capabilities; the collective behavior emerging from the self-organized interactions between the many robots of a swarm allow it to solve complex tasks (Beni, 2004; Sahin, 2004). To date, robot swarms have been demonstrated to solve tasks such as aggregation (Gauci et al., 2014), coordinated movement (Virágh et al., 2014), transportation of objects (Wang and Schwager, 2016), self-assembly (Rubenstein et al., 2014; Mathews et al., 2017), collective construction of structures (Werfel et al., 2014), and decentralized consensus formation (Schmickl and Crailsheim, 2008; Valentini et al., 2016).

Despite the variety of movement-centric and simple cognitive tasks that robot swarms have been demonstrated to perform (Bayındır, 2016), they continue to function largely as demonstration platforms in carefully controlled laboratory environments (Schranz et al., 2020), unable to transition to realistic application scenarios due to the following challenges: **Difficulties maintaining a high-density swarm:** A common feature of existing swarm robotic systems is the underlying assumption that the robots of the swarm act in close proximity of each other. Inter-robot distances of existing swarms are typically around 1–10 body lengths, both in indoor (Rubenstein et al., 2014; Pickem et al., 2017), and outdoor (Duarte et al., 2016; Zoss et al., 2018) environments. *Densely packed robot swarms* are inspired by social insect colonies, and rely on inter-robot physical interactions to complete their task. However, in employing such swarms in real-world outdoor applications encompassing large areas, the end-user faces a number of challenges involving the deployment and maintenance of such large numbers of robots during the mission. The recovery of the swarm post-mission is also problematic, particularly considering the high environmental cost of unrecovered robots. Furthermore, densely packed swarms are more likely to physically disrupt the other mission-participants, such as emergency workers in search and rescue operations.

**Constraints on inter-robot communication:** In most robot swarms, inter-robot coordination is reliant on an uninterrupted access to situated, close-range communication of coordination messages between robots (Duarte et al., 2016; Mathews et al., 2017, 2019; Garattoni and Birattari, 2018; Albani et al., 2019). However, in real-world scenarios robot swarms may face a number of challenges in exchanging coordination messages across the swarm. Swarms would be required to share communication channels with other participants in a mission, with high-bandwidth wireless channels most likely being reserved for human operators. Additionally, due to regulatory imposed channel-specific limitations on the communication duty-cycle (i.e., the proportion of time the transmitter is sending messages) (Semtech, 2015; Bor and Roedig, 2017), the robots of the swarm may also expect significant latency in receiving coordination messages.

**Restricted mobility and low endurance of robot platforms:** Most commercially available swarm robot platforms are designed to be operated over short distances (i.e., limited endurance) in carefully controlled indoor laboratory environments. This is particularly the case for swarms of ground robots that are typically constrained to operate on smooth, leveled surfaces such as table-tops (Mondada et al., 2009; Chamanbaz et al., 2017; Jones et al., 2018). Furthermore, low-cost outdoor platforms typically offer low autonomy and endurance, and are not thoroughly tested, compared to more costly alternatives.

In summary, despite the desirable characteristics of robustness and flexibility observed in collective systems in nature (Camazine et al., 2003), robot swarms inspired by such systems remain ill suited for realistic application scenarios. In mimicking the densities and coordination strategies of swarms in nature, swarm robotics faces a number of technological challenges relating to materials and their fabrication, power-efficiency, and battery-technologies for developing small-scale robots of a swarm that are compliant and autonomous in manners similar to their biological counterparts (Yang et al., 2018). Therefore, for robot swarms to be employed in realistic application scenarios, swarm technologies need to be reconceptualized.

In this paper, we propose the concept of *sparse swarms*, where the group of robots interact while (i) not being in close proximity to each other, and/or (ii) it is not possible for information to rapidly propagate within the group. Sparse swarms could be particularly relevant in application scenarios, where the robots are operating in the order of 1, 000 body lengths apart under sporadic low-bandwidth communication constraints. In such scenarios, the robots would be likely be required to coordinate their activities via informational interactions rather than physical interactions.

## 2. Related Concepts

This section notes similarities and differences between sparse swarms and two related concepts, cloud robotics and multirobot systems.

### 2.1. Cloud Robotics

In the domain of cloud robotics, robots separated by large distances perform some tasks, for example, grasping objects, while storing and sharing task-critical information over a “cloud” (Beetz et al., 2011; Kehoe et al., 2015; Wan et al., 2016). This is realized via machine-to-cloud (M2C) and/or machine-to-machine (M2M) communications (Hu et al., 2012). In both cloud robotics and sparse swarms, robots may rely on long-range interactions, for example, to share and learn from each others' experiences. However, while cloud-linked robots work on independent tasks in different environments, sparse swarm robots work on a common task in a shared environment, which requires them to coordinate their activities. Moreover, cloud-linked robots rely on costly external infrastructure—Internet connections providing high-bandwidth, low-latency communication with cloud services— which may not be available to sparse swarms deployed in real-world scenarios, for example, outdoors. The robots in a typical sparse swarm scenario are also likely to be less expensive than those in a typical cloud robotics scenario.

### 2.2. Multirobot Systems

While any robot swarm can be considered a multirobot system, the former term is usually preferred where a system comprises a relatively homogeneous group of robots, typically a dozen or more, which are unable to solve a given task efficiently on their own, but coordinate their activities, by exploiting only information that they can locally obtain, in the absence of global infrastructure (Sahin, 2004). With sparse swarms we consider groups of robots that are more sparsely distributed than present robot swarms, and even most multirobot systems (Chamanbaz et al., 2017). The high cost of currently available outdoor multirobot platforms prevents their adoption in robot swarms^1^. Moreover, many implementations of outdoor multirobot systems lack a fully decentralized, fault-tolerant control architecture, with the robots receiving instructions from a central planning/coordination node (Tardioli et al., 2016; Weinstein et al., 2018).

Some studies have focused on multirobot systems operating in communication-constrained environments (Amigoni et al., 2017; Tardioli et al., 2019). One approach is using some robots to physically deliver information to within communication range of other robots (Ducatelle et al., 2014; Cesare et al., 2015). Another approach is using some robots to form multi-hop communication chains, allowing for rapid propagation of information beyond the communication range of individual robots (Nouyan et al., 2009; Tardioli et al., 2010; Pei et al., 2013; Luo et al., 2019). Yet another approach is for the robots to reestablish contact, for example, periodically, at a priori known locations (Hollinger and Singh, 2012; Kantaros and Zavlanos, 2017) or using search (Banfi et al., 2018; Vandermeulen et al., 2018). Some of these approaches rely on a priori knowledge regarding how well robots can communicate between any two points in the environment (Amigoni et al., 2017; Banfi et al., 2018; Vandermeulen et al., 2018), which makes their application in real-world scenarios challenging.

## 3. Conceptualizing a Sparse Swarm

In the following, we describe two alternative characterizations of the sparse swarm concept. In both cases, we consider a swarm of *n* robots, *S* = {1, 2, …, *n*}.

### 3.1. Constraints on Inter-robot Proximity

In a sparse swarm, it would be costly for the robots to get into close proximity of each other (e.g., 10 body lengths away). To formalize this idea, we examine the swarm from a given time step, *k*_0_≥0, during the mission, for example, its start, *k*_0_ = 0. We refer to the swarm as *sparse* at time step *k*_0_ if the following condition is satisfied by a typical robot, *i*∈*S*:

(1) $$\begin{array}{r} {\mathrm{cost}}_{i}\left(\text{“move to nearest neighbor”},k_{0}\right)>>\\ {\mathrm{cost}}_{i}\left(\text{“perform typical operation”},k_{0}\right), \end{array}$$

where >> is defined as “at least one order of magnitude greater than,” and cost_*i*_ is a function that defines the *cost* for robot *i* to perform a given task at a given time. The cost could reflect the time taken, or energy expended, to complete the task. It would depend on the robot's capabilities and the environment the swarm resides in. What constitutes a “typical” operation would depend on the application scenario. For example, task “*perform typical operation”* could involve collecting a physical sample, or moving to the next waypoint. Task “*move to nearest neighbor”* could involve moving directly to the robot's nearest neighbor, or moving along a path of minimal cost.

Equation (1) suggests that for the typical robot in a sparse swarm, it may be prohibitively expensive to get into close proximity of another robot. The definition is sufficiently flexible to allow for occasional close encounters among some members of the swarm.

### 3.2. Constraints on Inter-robot Coupling

In a sparse swarm, it would not be possible for information to propagate rapidly to all of its members. To formalize this idea, we examine the swarm from a given time step, *k*_0_ ≥ 0, during the mission. Let **x**_*i*_[*k*] denote the state of robot *i* ∈ *S* at time step *k*. A robot's state could reflect its external configuration (e.g., pose) as well as its internal configuration (e.g., behavioral state, battery level). Let **z**_*i*_[*k*] denote the measurements that robot *i* ∈ *S* obtains at time step *k*. Let ${\tilde{\text{z}}}_{i}^{\left(j\right)}\left[k\right]$ denote the corresponding measurements that robot *i* would obtain had robot *j* not been present in the environment at time step *k*, and had all modifications that robot *j* made to the environment on or after time step *k*_0_ been discarded. By default, we assume that a robot's state transition function is affected by noise. Let *P*(**x**_*i*_[*k*]) denote the state distribution of robot *i* at time step *k* ≥ *k*_0_. For *k* > *k*_0_, let *A*[*k*] be the *n* × *n* matrix with

(2) $$\begin{array}{c} A_{i,j}\left[k\right]=\\ \left\{ \begin{array}{cc} 1, & P\left({\text{x}}_{i}\left[k\right]|{\text{x}}_{i}\left[k-1\right],{\text{x}}_{j}\left[k-1\right],{\text{z}}_{i}\left[k-1\right],{\text{z}}_{j}\left[k-1\right]\right)\\ \neq P\left({\text{x}}_{i}\left[k\right]|{\text{x}}_{i}\left[k-1\right],{\tilde{\text{z}}}_{i}^{\left(j\right)}\left[k-1\right]\right);\\ 0, & \text{otherwise.} \end{array}\right. \end{array}$$

Term *P*(**x**_*i*_[*k*]|**x**_*i*_[*k* − 1], **x**_*j*_[*k* − 1], **z**_*i*_[*k* − 1], **z**_*j*_[*k* − 1]) represents the conditional probability distribution of the state of robot *i* at time step *k* when the states and measurements of robots *i* and *j* are known at time step *k* − 1. It may depend on additional information, such as the environment, which is not explicitly represented here. Term $P\left({\text{x}}_{i}\left[k\right]|{\text{x}}_{i}\left[k-1\right],{\tilde{\text{z}}}_{i}^{\left(j\right)}\left[k-1\right]\right)$ represents the corresponding distribution under the assumption that robot *j* and all of its modifications made to the environment since time step *k*_0_ are currently discarded. If such “removal” of robot *j* would influence the conditional state distribution of robot *i* at time step *k*, the corresponding element of the matrix, *A*_*i, j*_[*k*], is 1, otherwise 0. Matrix *A* hence describes the possible interactions between all pairs.^2^ The couplings are directional. In other words, *A*_*i, j*_[*k*] = 1 does not imply *A*_*j, i*_[*k*] = 1. We assume that *A*_*ii*_ = 1 for all *i*, as robot *i*, once removed, would no longer have a well-defined state.

For τ ∈ {1, 2, … }, let

(3) $$\begin{array}{r} D\left(\tau \right)=\overset{k_{0}+\tau }{\underset{k=k_{0}+1}{\prod }}A\left[k\right]. \end{array}$$

In other words, matrix *D*(τ) is a product of matrices, which models the dependencies between pairs of robots within time period τ, starting from *k*_0_. Intuitively we consider all robots to be fully independent at time *k*_0_, that is, we discard the whole history of interactions up to time *k*_0_. Note that if a robot *i* influenced robot *j*, and robot *j* influenced robot *l* thereafter, then robot *i* influenced robot *l* as well.

Let

(4) $$\begin{array}{r} \tau_{min}={\text{argmin}}_{\tau }(D\left(\tau \right)\text{is not sparse}), \end{array}$$

where a matrix is considered sparse if half or more of its elements are zero. In other words, τ_*min*_ reflects the time it takes for information to propagate within the swarm. In particular, it denotes the earliest time after which robot *i* could have influenced robot *j* for the majority of pairs, (*i, j*) ∈ *S* × *S*. In the following, we assume that τ_*min*_ is finite. If τ_*min*_ = ∞, we would not refer to *S* as a swarm.

We refer to the swarm as *sparse* at time step *k*_0_ if

(5) $$\begin{array}{r} \tau_{min}=\Omega \left(n\right), \end{array}$$

that is, if τ_*min*_ is “at least as large as a constant times [*n*] for all large *n*” (Knuth, 1976). In other words, the time it takes for information to propagate grows at least linearly with the number of robots in the swarm.

A broad range of interactions can be captured using Equation (3). If the state of a robot described its position, an interaction could involve one robot pushing another robot, whether deliberately, or not. An interaction could involve one robot approaching a second robot, unless the presence of the second robot did not inform the choice of motion of the first. An interaction could involve a robot changing its state due to receiving a message by another robot. An interaction could involve a robot changing its state due to encountering a modification to the environment that was made by another robot. This latter form of interaction is commonly referred to as stigmergic communication.

The above two criteria are meant to complement each other. Where a swarm system is investigated in a concrete situation, the *constraints on inter-robot proximity* criterion can be used, taking into account the costs for a typical operation and that to reach the nearest neighbor. Where a swarm system is investigated over an infinite set of situations, involving groups of arbitrary size, the *constraints on inter-robot coupling* criterion can be used. This allows to evaluate the Ω(*n*) expression, which cannot be evaluated for swarms of constant size.

**Figure 1:**
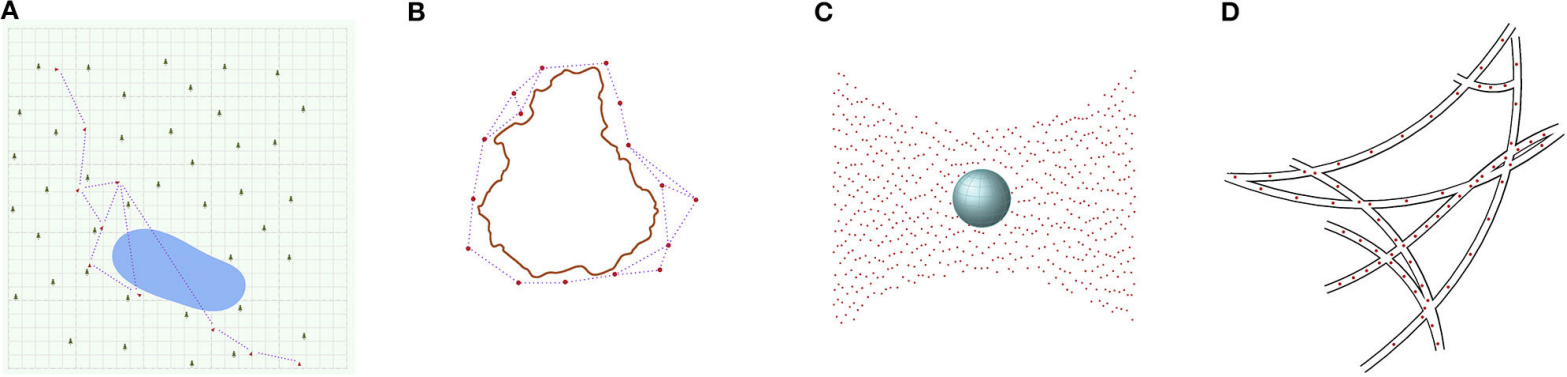
Application scenarios involving sparse swarms. A swarm of ten rovers (red markers) is tasked to monitor a 25 km^2^ forest ground. The rovers travel from South West to North East through the forest (depicted by tree symbols and green background), and the rovers that encountered the lake (depicted in blue) need to find a detour around it, leading to a concentration of rovers westwards of the lake **(A)**. A swarm of 16 surface vehicles is tasked to monitor the costal waters at the perimeter of an island of size 30 × 35 km^2^ (outlined in brown), which involves station-keeping around the island **(B)**. In both **(A,B)**, the communication links (indicated by dotted lines) are intermittent for the moving robots, due to signal attenuation by features of the environment. Free line-of-sight across the lake (in **A**) enhances the communication range. A swarm of 445 satellites self-organize into a 3-D spatial pattern providing continuous coverage for high-resolution imaging **(C)**. A swarm of around 100 microrobots search for blockages in a vascular network, using stigmergic interactions for coordination **(D)**.

Figure 1 illustrates the sparse swarm concept in four concrete situations, reflecting a range of application scenarios. In the first scenario, a group of 10 ground rovers operate in a squared forest region of side length 5 km. A typical operation for a ground rover may be to extract and store a sample of soil, which may take 30 s of time. The (median) distance to its nearest neighbor is 261 m. Assuming a terrain that allows the robot to move with an average speed of 0.2 m/s, the (median) time to move to the nearest neighbor would be 1,305 s. In the second scenario, a group of 16 unmanned surface vessels monitor the perimeter of an island of size 35 km North-to-South and 30 km East-to-West. A typical operation for a surface vehicle may be to maintain its position along the perimeter, which would require significantly less energy than that required for the vehicle to sail a (median) distance of 6.8 km to its nearest neighbor. Such station-keeping mission scenario for a swarm of surface vehicles may also be extrapolated to 3-D for underwater, aerial and space environments, requiring larger sized swarms that are still sparse. Given the constraints on inter-robot proximity, above groups could be considered sparse swarms. Moreover, they are characterized by predominantly linear inter-robot communication networks. As the swarms would have to encompass larger environments, they would have to be proportionally larger in size, and the time it takes for information to propagate within the swarm would increase linearly with the number of robots. This would thus satisfy our constraint for inter-robot coupling for sparse swarms. An interesting scenario are in-body applications where using a dense swarm of robots may be too invasive. Instead, a sparse swarm of microrobots could be used, for example, to explore the vascular network for blockages. In such applications, the microrobots may coordinate their response using stigmergic interactions.

## 4. Forest Application Scenario

In this section, we discuss the aforementioned forest application scenario, including the associated challenges in realizing the concept of a sparse swarm. Consider a sparse swarm of 10 terrestrial robots (i.e., rovers) tasked with monitoring a large tract of 25 km^2^ forest ground (Figure 1A). The robots are deployed at one end of the forest equidistant to each other, and are tasked with sweeping through the forest in a quasi line-formation; quasi as the robots are traversing on uneven terrain and are consequently unable to maintain a constant velocity across the swarm. The proposed scenario allows us to assess the following challenges: (i) the mechanical design of platform hardware in terms of its capability to efficiently traverse difficult terrain; (ii) algorithms for terrain perception and robot locomotion over difficult terrain; (iii) the selection of a long-range inter-robot communication technology for a forest environment; and (iv) the design of the decentralized coordination strategies for the sparse swarm. We detail these challenges and introduce our ongoing work to address them.

### 4.1. Robot Platform Design

Although different forest environments may present different sets of requirements, the latter have typically the following ones in common: (i) the ability of the rover to progress fast through simple terrain; (ii) the ability to either overcome or avoid obstacles in its path, and (iii) long endurance. The need to traverse long distances requires energy efficient mobility, which is easiest achieved by rolling. For practicality, our interest is in rovers that are small enough to fit in a backpack. The size of obstacles the rover will be able to overcome is accordingly limited. Furthermore, the overall cost of each rover should be low enough that sizeable swarms are practical. In the context of these constraints the robot platform needs to address the challenges of mobility and communication.

#### 4.1.1. Mechanical Design for Mobility

**Figure 2:**
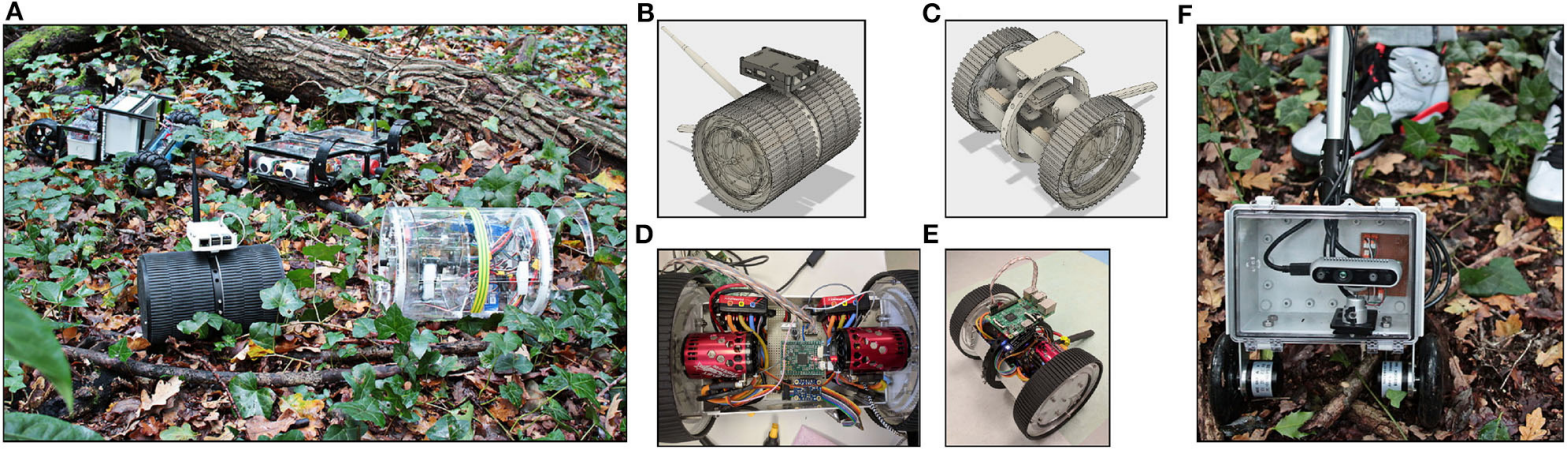
Hardware platforms for the forest environment. Aside from four-/six-wheel drive, and tracks, other locomotion concepts are also investigated for their suitability on forest ground **(A)**. The torque available to platforms with brushless motors (e.g., **B–E**) is helpful for tackling the ubiquitous small obstacles typical for this environment. Additional data for computer vision development is collected with a manual rig **(F)**. Depth and color images are recorded with a global shutter camera (D435i, www.intel.com) to a laptop in a backpack. Meta data is collected from the camera's inertial measurement unit, rotary encoders on the wheels, and a GPS. A mobile phone mounted on the telescopic push rod gives remote access to the laptop.

A rover that is well-adapted to the forest environment will provide a good trade-off between the ability to climb over obstructions to avoid detours at the reduced endurance that results from the extra weight of this climbing ability. We use an iterative design strategy where data on energy consumption and mobility is gathered by teleoperated prototype platforms (Figures 2A–E). In addition to the on-board data collection, telemetry provides real-time feedback during such test runs to improve our understanding of what obstacles can be tackled by a particular rover design, what are suitable approaches to do so, and what is the energy expended for a particular path.

#### 4.1.2. Hardware for Communication

Communication is the only form of direct interaction that is considered here. It needs to be scalable to many units and work over long range even with antennas located close to the ground. In many application scenarios the intra-swarm communication cannot be prioritized over other services. For radio communication these requirements point to limits in the frequency spectrum and transmission power that in combination with the range requirement lead to low channel capacity. Such a low capacity channel could be established over satellite communication or over text messages transmitted in a mobile phone network. However, a solution that does not rely on infrastructure is preferred, both from a cost and from an availability perspective. In the field of sensor networks and internet of things ultra-high-frequency radio technologies have recently come to the fore that aim for long range communication with low power requirements, scalability to several thousands nodes, and low hardware cost. One of these technologies, called long-range radio (LoRa), is particularly attractive in the present context of rovers. Our preliminary exploration of the suitability of LoRa for rovers operating at forest ground indicate that several hundred meters communication range is realistic at about 60 bytes per second. This is the case, even in the highly attenuating forest environment and with the ground plane effect inherent in a low antenna position (tip of antenna 17 cm above ground).

### 4.2. Locomotion on Difficult Terrain

Navigating off-trail in a forest environment is a challenging task and an open problem in the area of field robotics (Yang et al., 2018). The robots are required to assess their traversability on a priori unknown terrains in their proximity, relying solely on onboard sensors under varying lighting and weather conditions, where GPS signal localization may not always be available. The problem is made further difficult by the varying nature of traversability; the traversability of a robot on a terrain depends not only on the innate characteristics of the terrain, but also on the dynamics of interaction between the robot and the terrain, which itself is susceptible to change (e.g., from a thick layer of mud stuck on the left side of a six-wheeled robot, or a damaged leg sustained by a quadruped robot).

Many studies have investigated terrain traversability for robot navigation algorithms in off-road environments, pioneered by the DARPA PerceptOR (Krotkov et al., 2007) and later the DARPA Learning Applied to Ground Vehicles (LAGR) programs (Huang et al., 2009a,b). The approaches developed for terrain traversability analysis use exteroceptive sensory information such as geometry-based and appearance-based features, as well as proprioceptive sensory information (Papadakis, 2013), and typically employ near-to-far type of learning algorithms (Bagnell et al., 2010) to predict traversable terrain for the robot. However, the robots employed in such off-road situations are relatively large (e.g., the DARPA LAGR vehicle was over 1 m in length and weighed around 100 kg), and equipped with expensive sensors such as radar, 2D lidar and multiple stereo cameras for off-road navigation (Jackel et al., 2006; Zhou et al., 2012; Milella et al., 2015; Santamaria-Navarro et al., 2015). In comparison, our small-scale low-cost robots running off-trail in the forest are faced with bigger challenges: Almost everything is an obstacle, and due to their small size the robots are much more likely to topple over. The development of computationally inexpensive computer vision and machine learning algorithms for the robots to efficiently locomote over a priori unknown terrains is part of our ongoing effort to realize our sparse swarm.

In addressing the traversability challenge we are in the process of developing a forest environment RGBD data-set, using a two-wheel mobile sensor platform (Figure 2F). The platform comprising an Intel D435i depth camera including an IMU, left and right wheel encoders, and GPS, is to be pushed manually along various off-trail “paths.” Our developed data-set is to be employed to train a depth estimation model, to predict depth with RGB image data from a monocular camera.

#### 4.2.1. Terrain Traversability for a Single Robot

Using the depth-estimation model, the robots of the sparse swarm are required to learn closed-loop policies to efficiently traverse across different terrains. Forest terrain the robot may have to overcome include wet leaves on the forest floor, ditches with varying inclinations, muddy tracks and fallen tree branches. Challenges involved in learning locomotion behaviors for such terrain include investigating suitable representations for a closed-loop policy, characterizing metrics to estimate success of a policy in overcoming terrain, and accounting for progress between trials in evaluating multiple policies episodically on the robot. Trial-and-error based algorithms for rapid behavior adaptation (e.g., see Cully et al., 2015) appear to be a promising approach to begin addressing these challenges.

#### 4.2.2. Collaborative Learning Across the Swarm

The available LoRa communication channel may be employed by the swarm for collaborative learning of traversable terrain in the forest environment. In such a transfer learning scenario, the robots of the swarm share information on their experiences traversing different terrains. Information shared may comprise metrics providing situational information on robot-terrain interaction, for instance energy consumption statistics, and the stability of the robot in traversing the terrain. Policies employed by robots to traverse terrain may also be shared, for recipient robots to bootstrap their exploration of new locomotion behaviors to adapt to changes in their proximal terrain. Additionally, in forest environments, some a priori unknown terrains may be unsafe for the robot to traverse over. The discovery of such terrains by the swarm may be accomplished by learning with “deliberative” catastrophic failure. Herein, the swarm may vote for one or a few robots to attempt to traverse over potentially hazardous terrain and share the resulting traversability information generated with the rest of the swarm.

### 4.3. Coordination in Communication-Constrained Environments

In the forest application scenario, the robots assume a linear formation that moves across a defined region. In a simple linear formation, the robots would occupy equidistant points on a line segment; each robot, bar the ones at the two ends, would have two neighbors. An alternative linear formation would place the robots alternatingly onto two parallel lines such that they form equilateral triangles; each robot within the formation would have four equidistant neighbors. To ease deployment, the robots could determine their order within the formation at run-time, for example, using their unique identifiers. While linear formations lend themselves for tasks such as coordinated search and coverage (Durham et al., 2012; Kolling et al., 2018), our scenario is particularly challenging, because the robots will be unable to interact with their neighbors for most of the time. Moreover, they do not know in advance the terrain to be encountered. This makes it difficult to predict individual progress. Some robots may have to take a detour of several hundreds of meters after discovering that a floodplain ahead of them is not traversable. To cope efficiently with these challenges, the robots need to move even when having had no recent contact with any neighbor. Yet, they should prevent the overall formation from becoming disconnected indefinitely. The robots could generate waypoints, and use the potential field method to approach them while avoiding obstacles. New way points could be suggested in an attempt to move the formation forward, and to repair it. The robots would use beliefs regarding their neighborhood, that is, which robots are present and their locations. Algorithms that allow robots to reestablish contact with lost, and potentially immobilized, members of the group could be considered (Banfi et al., 2018; Vandermeulen et al., 2018).

## 5. Discussion

In this perspective article, we have highlighted the challenges that prevent most swarm robotic systems from transitioning to real-world applications. At present, robot swarms typically operate in highly controlled indoor laboratory environments. They are frequently interacting with each other, which is facilitated by their spatial proximity (e.g., 10 body lengths). Consequentially, such swarms are impractical for many real-world applications, in particular those, requiring the robots to act jointly over large distances (e.g., 1,000 body lengths). To address this problem, we have proposed the *sparse swarm* concept, which focuses on robot swarms that self-organize despite severe constraints regarding inter-robot proximity and coupling. Moreover, we have illustrated its use in a forest application scenario.

The sparse swarm concept opens up a number of theoretical questions. While sparse swarms are robot swarms, they are subject to additional constraints on inter-robot proximity and inter-robot coupling. A question to investigate is how the performance for a given swarm changes as these constraints are progressively enforced. A related question is how the minimal number of robots to exhibit self-organization changes as the constraints increase. For example, will swarms degenerate once the time for information to propagate is no longer polynomially bounded with the number of robots? Another question relates to the types of interactions. Where members of sparse swarms interact solely via non-situated communication, can they still spatially organize, for example, by sharing information on how to interact with the environment? And given the lack of spatial proximity, would the members of sparse swarms be required to encounter a similar set of environmental features (which could be empty) to exhibit self-organization? A further question relates to whether sparse swarms could be realized at all scales, with their members ranging in size from hundreds of meters (e.g., fleets of container ships) to micrometers (e.g., robot swarms within the human body).

For a sparse robot swarm to solve real-world problems in a land, sea, air or space environment, the individual robots are likely to require a high degree of autonomy and the ability to travel and to communicate over long distances. Depending on the environment and the task at hand the practical challenges to achieving the required capabilities differ. In environments that allow for energy harvesting (e.g., consider solar-powered aerial drones or autonomous sailboats), endurance is not limited by power, but by the device life-time. As a consequence of the much increased deployment time across the sparse swarm, rare events can no longer be ignored. For such a system, what general strategies that broaden the ability of a system to recover from unforeseen situations (e.g., Cully et al., 2015) can be developed? Moreover, in many sparse swarm scenarios the channel capacity for communicating within the swarm is severely restricted (e.g., robots operating under water). How can the mismatch between the amount of data available from local sensors and the amount of data that can be received from others robots be reconciled for effective learning?

In conclusion, directly mimicking the densities and associated coordination strategies of natural swarms may be impractical for applications that require groups of robots to cover outdoor areas that are very large relative to their own size. We postulate that for these applications, swarm technologies need to be reconceptualized for robots to coordinate over large distances. Such coordination without any physical inter-robot interaction would require higher autonomy from individual robots of the swarm. Robots of the swarm would also require to traverse large distances to complete their mission, thus requiring low-cost, high-endurance hardware platforms. With this perspective article, we invite the robotics community to address the various challenges to bring sparse swarms to fruition.

## Author Contributions

All authors listed have made a substantial, direct and intellectual contribution to the work, and approved it for publication.

## Conflict of Interest

The authors declare that the research was conducted in the absence of any commercial or financial relationships that could be construed as a potential conflict of interest.

## Footnotes

^1^Note that we are not postulating to reduce the number of robots in swarms—merely their density.

^2^Note that if the states of two robots are correlated this would not necessarily imply that an interaction took place. It could be that both robots independently discovered a same environmental feature.

## References

[B1] Albani D. Manoni T. Arik A. Nardi D. Trianni V. (2019). “Field coverage for weed mapping: toward experiments with a UAV swarm,” in International Conference on Bio-inspired Information and Communication (Pittsburgh, PA: Springer), 132–146. 10.1007/978-3-030-24202-2_10

[B2] Amigoni F. Banfi J. Basilico N. (2017). Multirobot exploration of communication-restricted environments: a survey. IEEE Intell. Syst. 32, 48–57. 10.1109/MIS.2017.4531226

[B3] Bagnell J. A. Bradley D. Silver D. Sofman B. Stentz A. (2010). Learning for autonomous navigation. IEEE Robot. Autom. Mag. 17, 74–84. 10.1109/MRA.2010.936946

[B4] Banfi J. Basilico N. Amigoni F. (2018). Multirobot reconnection on graphs: problem, complexity, and algorithms. IEEE Trans. Robot. 34, 1299–1314. 10.1109/TRO.2018.2830418

[B5] Bayındır L. (2016). A review of swarm robotics tasks. Neurocomputing 172, 292–321. 10.1016/j.neucom.2015.05.116

[B6] Beetz M. Civera J. D'Andrea R. Elfring J. Galvez-lopez D. (2011). RoboEarth: a world wide web for robots. IEEE Trans. Robot. Autom. 6, 69–82. 10.1109/MRA.2011.941632

[B7] Beni G. (2004). “From swarm intelligence to swarm robotics,” in International Workshop on Swarm Robotics (Berlin: Springer), 1–9. 10.1007/978-3-540-30552-1_1

[B8] Bor M. Roedig U. (2017). “LoRa transmission parameter selection,” in 2017 13th International Conference on Distributed Computing in Sensor Systems DCOSS (Los Alamitos, CA: IEEE Computer Society). 10.1109/DCOSS.2017.10

[B9] Camazine S. Deneubourg J.-L. Franks N. R. Sneyd J. Bonabeau E. Theraula G. (2003). Self-Organization in Biological Systems. Princeton, NJ: Princeton University Press.

[B10] Cesare K. Skeele R. Yoo S.-H. Zhang Y. Hollinger G. (2015). “Multi-UAV exploration with limited communication and battery,” in 2015 IEEE International Conference on Robotics and Automation (ICRA) (Seattle, WA: IEEE), 2230–2235. 10.1109/ICRA.2015.7139494

[B11] Chamanbaz M. Mateo D. Zoss B. M. Tokić G. Wilhelm E. Bouffanais R. (2017). Swarm-enabling technology for multi-robot systems. Front. Robot. AI 4:12 10.3389/frobt.2017.00012

[B12] Cully A. Clune J. Tarapore D. Mouret J.-B. (2015). Robots that can adapt like animals. Nature 521:503. 10.1038/nature14422 26017452

[B13] Duarte M. Costa V. Gomes J. Rodrigues T. Silva F. Oliveira S. M.. (2016). Evolution of collective behaviors for a real swarm of aquatic surface robots. PLoS ONE 11:e0151834. 10.1371/journal.pone.0151834 26999614 PMC4801206

[B14] Ducatelle F. Di Caro G. A. Förster A. Bonani M. Dorigo M. Magnenat S. (2014). Cooperative navigation in robotic swarms. Swarm Intell. 8, 1–33. 10.1007/s11721-013-0089-4

[B15] Durham J. W. Franchi A. Bullo F. (2012). Distributed pursuit-evasion without mapping or global localization via local frontiers. Auton. Robots 32, 81–95. 10.1007/s10514-011-9260-1

[B16] Garattoni L. Birattari M. (2018). Autonomous task sequencing in a robot swarm. Sci. Robot. 3:eaat0430 10.1126/scirobotics.aat0430 33141726

[B17] Gauci M. Chen J. Li W. Dodd T. J. Groß R. (2014). Self-organized aggregation without computation. Int. J. Robot. Res. 33, 1145–1161. 10.1177/0278364914525244

[B18] Hollinger G. A. Singh S. (2012). Multirobot coordination with periodic connectivity: theory and experiments. IEEE Trans. Robot. 28, 967–973. 10.1109/TRO.2012.2190178

[B19] Hu G. Tay W. P. Wen Y. (2012). Cloud robotics: architecture, challenges and applications. IEEE Netw. 26, 21–28. 10.1109/MNET.2012.6201212

[B20] Huang W. Grudic G. Matthies L. (2009a). Special issue: special issue on LAGR program, part I. J. Field Robot. 26, 1–113. 10.1002/rob.v26:1

[B21] Huang W. Grudic G. Matthies L. (2009b). Special issue: special issue on LAGR program, part II. J. Field Robot. 26, 115–240. 10.1002/rob.20280

[B22] Jackel L. D. Krotkov E. Perschbacher M. Pippine J. Sullivan C. (2006). The DARPA LAGR program: goals, challenges, methodology, and phase i results. J. Field Robot. 23, 945–973. 10.1002/rob.20161

[B23] Jones S. Studley M. Hauert S. Winfield A. F. T. (2018). A two teraflop swarm. Front. Robot. AI 5:11 10.3389/frobt.2018.00011 PMC7805610 33500898

[B24] Kantaros Y. Zavlanos M. M. (2017). Distributed intermittent connectivity control of mobile robot networks. IEEE Trans. Autom. Control 62, 3109–3121. 10.1109/TAC.2016.2626400

[B25] Kehoe B. Patil S. Abbeel P. Goldberg K. (2015). A survey of research on cloud robotics and automation. IEEE Trans. Autom. Sci. Eng. 12, 398–409. 10.1109/TASE.2014.2376492

[B26] Knuth D. E. (1976). Big omicron and big omega and big theta. SIGACT News 8, 18–24. 10.1145/1008328.1008329

[B27] Kolling A. Kleiner A. Carpin S. (2018). Coordinated search with multiple robots arranged in line formations. IEEE Trans. Robot. 34, 459–473. 10.1109/TRO.2017.2776305

[B28] Krotkov E. Fish S. Jackel L. McBride B. Perschbacher M. Pippine J. (2007). The DARPA PerceptOR evaluation experiments. Auton. Robots 22, 19–35. 10.1007/s10514-006-9000-0

[B29] Luo W. Sycara K. (2019). “Minimum k-connectivity maintenance for robust multi-robot systems,” in 2019 IEEE/RSJ International Conference on Intelligent Robots and Systems (IROS) (Macau: IEEE), 7370–7377.

[B30] Mathews N. Christensen A. L. O'Grady R. Mondada F. Dorigo M. (2017). Mergeable nervous systems for robots. Nat. Commun. 8:439 10.1038/s41467-017-01622-0 28900125 PMC5595853

[B31] Mathews N. Christensen A. L. Stranieri A. Scheidler A. Dorigo M. (2019). Supervised morphogenesis: exploiting morphological flexibility of self-assembling multirobot systems through cooperation with aerial robots. Robot. Auton. Syst. 112, 154–167. 10.1016/j.robot.2018.11.007

[B32] Milella A. Reina G. Underwood J. (2015). A self-learning framework for statistical ground classification using radar and monocular vision. J. Field Robot. 32, 20–41. 10.1002/rob.21512

[B33] Mondada F. Bonani M. Raemy X. Pugh J. Cianci C. Klaptocz A. (2009). “The e-puck, a robot designed for education in engineering,” in Proceedings of the 9th Conference on Autonomous Robot Systems and Competitions (IPCB: Instituto Politécnico de Castelo Branco), 59–65.

[B34] Nouyan S. Groß R. Bonani M. Mondada F. Dorigo M. (2009). Teamwork in self-organized robot colonies. IEEE Trans. Evol. Comput. 13, 695–711. 10.1109/TEVC.2008.2011746

[B35] Papadakis P. (2013). Terrain traversability analysis methods for unmanned ground vehicles: a survey. Eng. Appl. Artif. Intell. 26, 1373–1385. 10.1016/j.engappai.2013.01.006

[B36] Pei Y. Mutka M. W. Xi N. (2013). Connectivity and bandwidth-aware real-time exploration in mobile robot networks. Wireless Commun. Mobile Comput. 13, 847–863. 10.1002/wcm.1145

[B37] Pickem D. Glotfelter P. Wang L. Mote M. Ames A. Feron E. (2017). “The Robotarium: a remotely accessible swarm robotics research testbed,” in 2017 IEEE International Conference on Robotics and Automation (ICRA) (Singapore: IEEE), 1699–1706. 10.1109/ICRA.2017.7989200

[B38] Rubenstein M. Cornejo A. Nagpal R. (2014). Programmable self-assembly in a thousand-robot swarm. Science 345, 795–799. 10.1126/science.1254295 25124435

[B39] Sahin E. (2004). “Swarm robotics: From sources of inspiration to domains of application,” in International Workshop on Swarm Robotics (Berlin: Springer), 10–20. 10.1007/978-3-540-30552-1_2

[B40] Santamaria-Navarro À. Teniente E. H. Morta M. Andrade-Cetto J. (2015). Terrain classification in complex three-dimensional outdoor environments. J. Field Robot. 32, 42–60. 10.1002/rob.21521

[B41] Schmickl T. Crailsheim K. (2008). Trophallaxis within a robotic swarm: bio-inspired communication among robots in a swarm. Auton. Robots 25, 171–188. 10.1007/s10514-007-9073-4

[B42] Schranz M. Umlauft M. Sende M. Elmenreich W. (2020). Swarm robotic behaviors and current applications. Front. Robot. AI 7:36 10.3389/frobt.2020.00036 PMC7805972 33501204

[B43] Semtech (2015). LoRa Modulation Basics. Application Note AN1200.22, Semtech Corporation, Camarillo, CA.

[B44] Tardioli D. Mosteo A. R. Riazuelo L. Villarroel J. L. Montano L. (2010). Enforcing network connectivity in robot team missions. Int. J. Robot. Res. 29, 460–480. 10.1177/0278364909358274

[B45] Tardioli D. Riazuelo L. Sicignano D. Rizzo C. Lera F. Villarroel J. L. (2019). Ground robotics in tunnels: keys and lessons learned after 10 years of research and experiments. J. Field Robot. 36, 1074–1101. 10.1002/rob.21871

[B46] Tardioli D. Sicignano D. Riazuelo L. Romeo A. Villarroel J. L. Montano L. (2016). Robot teams for intervention in confined and structured environments. J. Field Robot. 33, 765–801. 10.1002/rob.21577

[B47] Valentini G. Ferrante E. Hamann H. Dorigo M. (2016). Collective decision with 100 kilobots: speed versus accuracy in binary discrimination problems. Auton. Agents Multi Agent Syst. 30, 553–580. 10.1007/s10458-015-9323-3

[B48] Vandermeulen I. Groß R. Kolling A. (2018). “Re-establishing communication in teams of mobile robots,” in 2018 IEEE/RSJ International Conference on Intelligent Robots and Systems (IROS) (Madrid: IEEE), 7947–7954. 10.1109/IROS.2018.8594460

[B49] Virágh C. Vásárhelyi G. Tarcai N. Szörényi T. Somorjai G. Nepusz T.. (2014). Flocking algorithm for autonomous flying robots. Bioinspir. Biomimet. 9:025012. 10.1088/1748-3182/9/2/025012 24852272

[B50] Wan J. Tang S. Yan H. Li D. Wang S. Vasilakos A. V. (2016). Cloud robotics: current status and open issues. IEEE Access 4, 2797–2807. 10.1109/ACCESS.2016.2574979

[B51] Wang Z. Schwager M. (2016). Force-Amplifying N-robot Transport System (Force-ANTS) for cooperative planar manipulation without communication. Int. J. Robot. Res. 35, 1564–1586. 10.1177/0278364916667473

[B52] Weinstein A. Cho A. Loianno G. Kumar V. (2018). Visual inertial odometry swarm: An autonomous swarm of vision-based quadrotors. IEEE Robot. Autom. Lett. 3, 1801–1807.

[B53] Werfel J. Petersen K. Nagpal R. (2014). Designing collective behavior in a termite-inspired robot construction team. Science 343, 754–758. 10.1126/science.1245842 24531967

[B54] Yang G.-Z. Bellingham J. Dupont P. E. Fischer P. Floridi L. Full R. (2018). The grand challenges of science robotics. Sci. Robot. 3:eaar7650 10.1126/scirobotics.aar7650 33141701

[B55] Zhou S. Xi J. McDaniel M. W. Nishihata T. Salesses P. Iagnemma K. (2012). Self-supervised learning to visually detect terrain surfaces for autonomous robots operating in forested terrain. J. Field Robot. 29, 277–297. 10.1002/rob.21417

[B56] Zoss B. M. Mateo D. Kuan Y. K. Tokić G. Chamanbaz M. Goh L. (2018). Distributed system of autonomous buoys for scalable deployment and monitoring of large waterbodies. Auton. Robots 42, 1669–1689. 10.1007/s10514-018-9702-0

